# Linking motor-related brain potentials and velocity profiles in multi-joint arm reaching movements

**DOI:** 10.3389/fnhum.2014.00271

**Published:** 2014-04-29

**Authors:** Julià L. Amengual, Josep Marco-Pallarés, Carles Grau, Thomas F. Münte, Antoni Rodríguez-Fornells

**Affiliations:** ^1^Cognition and Brain Plasticity Unit, Department of Basic Psychology, University of BarcelonaBarcelona, Spain; ^2^Bellvitge Biomedical Research Institute (IDIBELL), Hospitalet de LlobregatSpain; ^3^Neurodynamic Laboratory, Department of Psychiatry and Clinical Psychobiology, Universitat de BarcelonaBarcelona, Spain; ^4^Department of Neurology, University of LübeckLübeck, Germany; ^5^Institució Catalana de Recerca i Estudis AvançatsBarcelona, Spain

**Keywords:** motor related brain potentials, 3-D movement analyser, time-series analysis, kinematics, self-paced movement, motor activity

## Abstract

The study of the movement related brain potentials (MRPBs) needs accurate technical approaches to disentangle the specific patterns of bran activity during the preparation and execution of movements. During the last forty years, synchronizing the electromyographic activation (EMG) of the muscle with electrophysiological recordings (EEG) has been commonly ussed for these purposes. However, new clinical approaches in the study of motor diseases and rehabilitation suggest the demand of new paradigms that might go further into the study of the brain activity associated with the kinematics of movements. As a response to this call, we have used a 3-D hand-tracking system with the aim to record continuously the position of an ultrasonic sender attached to the hand during the performance of multi-joint self-paced movements. We synchronized time-series of position and velocity of the sender with the EEG recordings, obtaining specific patterns of brain activity as a function of the fluctuations of the kinematics during natural movement performance. Additionally, the distribution of the brain activity during the preparation and execution phases of movements was similar that reported previously using the EMG, suggesting the validity of our technique. We claim that this paradigm could be usable in patients because of its simplicity and the potential knowledge that can be extracted from clinical protocols.

## Introduction

Over the lasts 40 years, the electrophysiological brain activity (EEG) associated with the preparation and execution of movements has been widely described. The Bereitschaftspotential (BP) (Kornhuber and Deecke, 1965), also termed readiness potential, is a slow negativity starting 1.5–2 s before the onset of the movement that shows a wide scalp distribution being maximal over centro-parietal regions. In addition to the BP itself, a set of components related with to the preparation and the execution of movements has been identified, being known as movement-related brain potentials (MRBPs) (see Shibasaki and Hallett, 2006, for a review). Furthermore, during the preparation and execution of voluntary movements, a characteristic modulation of the oscillatory brain activity power within the beta (17–24 Hz) and the mu (8–13 Hz) bands has been largely described (Pfurtscheller and Aranibar, 1977, 1979; Pfurtscheller et al., 2003; Jurkiewicz et al., 2006).

In order to give a more fine-grained characterization of the neural sources of these potentials and the associated oscillatory brain activity, several studies have used the Laplacian transformed activity of the EEG obtaining the current source density (CSD) waveforms (Nunez, 2002; Carbonnell et al., 2004; Kayser et al., 2010; Meckler et al., 2010; Tenke and Kayser, 2012). This method allows evaluating the topographical distribution of the brain activity in terms of current sources and sinks through the scalp (Kayser and Tenke, 2006). The maps of activity generated by these CSD waveforms are sensitive to high spatial frequency changes of local cortical potentials due to reduced volume conduction form distant sources (Le et al., 1994). Additionally, it minimizes smearing effects as caused by the tissue transmission distortion (Perrin et al., 1989) and other possible artifacts (Kayser and Tenke, 2006). Particularly, it has been proposed that this transformation is especially useful in localizing the sources of activity in sensorimotor tasks (Tenke and Kayser, 2012).

Classical paradigms designed to study these MRBPs use the surface electromyographic (EMG) signal originated by one muscle or group of muscles recorded simultaneously to the EEG to measure the activation of these muscles while subjects repeat movements at self-pace rates (Cui et al., 1999; Ohara et al., 2006). This technique allows the identification of the movement onset as a rebound in the EMG signal, allowing to off-line epoch the EEG time-locked to the onset of each movement and the posterior averaging of these epochs. Additionally, this signal provides useful information about the strength of the muscular contraction, allowing the characterization of the components of the MRBPs as a function of the kinetic parameters of movement. However, only a few studies have focused on the association between these components with kinematic properties of movement (e.g., position, velocity and acceleration). Slobounov et al. (2000) reported an increase in the amplitude of the late component of the BP (the so-called *late BP* Shibasaki and Hallett, 2006) when the maximum degree of the index finger extension was achieved. In a pioneering study Kirsch et al. (2010) reported a positive correlation between the amplitude of the BP and both velocity and distance during the execution of goal-directed movements. In addition, they found that an increase of the target distance and the movement time were associated with the smoothness of the time-course of the BP. To this aim, these authors used a goal-directed movement paradigm and measured the movement performance using a 3D hand-tracking system to identify the movement onset instead of using the classical EMG signal. This procedure allowed them to establish a relationship between the internal forces of the movement (i.e., kinetics) and the external motion parameters as position and velocity (i.e., kinematics) to the electrical brain activity. Other studies have used the time series of velocity coupled with the EEG signal in order to develop brain computer interface (BCI) methods. In an outstanding study, Bradberry et al. (2010) estimated the trajectories of self-paced reaching movements by extracting associated patterns of EEG activity. They used a 3D tracking system to continuously extract the hand velocity coupled with the EEG signal and estimated the sources of brain activity that were more strongly involved in encoding the hand velocity using sLORETA. However, no studies to present have established a direct relationship between changes in the pattern of velocity during movement performance and the associated EEG activity at each stage of the movement.

We aimed to investigate the cross-relationship between the fluctuations of the velocity during the execution of natural self-paced movements and the concomitant EEG activity. Self-paced movements have the property of being self-initiated, that is, triggered by the internal decision of the subject instead of being externally triggered. In the present study we designed a paradigm that required participants to reach a target positioned at a given exact location using both arms through multi-joint arm reaching movements. During the task, the 3D spatial position of an ultrasound marker located on the hand was recorded using a hand-tracking system, thus obtaining the time series of the position of this marker. We synchronized this signal with that obtained from the EEG recordings with two different aims. First, we wanted to establish the onset of each movement using the derived time series of the spatial position (that is, the time series of velocity), which permitted epoching the EEG time-locked to this time-point as similarly done in previous studies with the EMG signal (Cui et al., 1999; Ohara et al., 2006). Second, we aimed to directly compare the time series of the velocity of the sender with the components of the MRBPs and their CSD transformed signal, determining a point-to-point relationship between the different phases of the movement execution and the concomitant brain activity. To our knowledge, no previous studies have addressed this issue, and we hypothesize that this novel manner to work with these components would allow finding patterns of neural and oscillatory activity related to variations of the velocity during movement performance. Finding similar results to those reported in studies using the EMG as movement-related signal would indicate the validity of this technique to study movement related brain activity. Also, because of the simplicity of our experimental design, our study highlights the potential of examining the brain activity associated with movement using the hand-tracking system in clinical protocols.

## Materials and methods

### Participants

Fifteen right-handed healthy volunteers participated in this experiment (8 women, mean age 26.51 ± 3.42 years). All participants were drug free and had no history of neurological diseases. They all gave written informed consent and were paid for their participation in the study. The study was approved by the ethics committee of the University of Barcelona and was conducted according to the Helsinki Declaration.

### Experimental procedure

**Figure 1:**
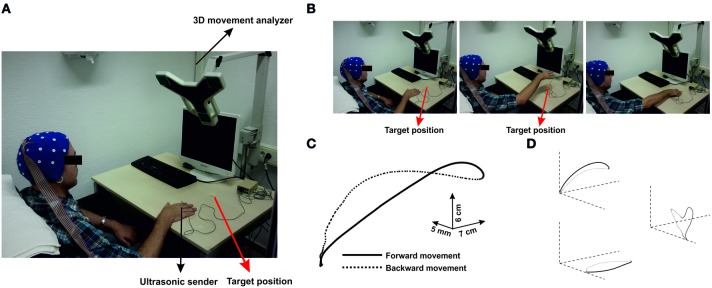
**(A)** Configuration of the experimental setup. Parcitipants sat in a confortable position in front of a table. An ultrasonic sender was located on the index finger of the active hand. A 3-D movement analyzer recorded the position of the sender during self-paced movements to the target position (red arrow). **(B)** Time-line of one representative trial. Each picture corresponds to different time points during movement (preparation, achievement of the maximum height and reaching the target). Movements were performed as multi-joint arm reaching through elbow-extensions. **(C)** Three-dimensional representation of the averaged time series of all movements performed by one representative participant. **(D)** Projections over the three planes are represented. Continuous black lines correspond to the forward movement, whereas black dashed lines correspond to the backward movement towards the initial position.

Participants sat in a comfortable chair. They rested their hand on the table surface, about 10 cm from the edge, with the index finger in extended position. They were asked to perform self-paced pointing movements reaching a white target plate located 20 cm in front of the starting point with their extended index finger (Figure 1). These movements consisted in elbow extensions, which might additionally involve other components as shoulder extension (see Figure 1B). Therefore, we will refer to these movements as multi-join reaching movements. The trajectory of these movements was parabolic-shaped, i.e., they were not to drag the arm on the table to achieve the target, and they had to perform the backward movement to the initial position as soon as the target was reached. Thus, movements were performed with a very sharp onset, starting out from muscular relaxation.

Importantly, no external cue was used to trigger the intention of the movement, so that subjects performed each movement on their own. They were asked to allow an interval of 7–10 s between each movement. In order to avoid horizontal eyes movement artifacts, subjects were instructed to fixate their gaze to the target cue during the whole task and not to blink from about 3 s before movement onset to around 4 s after completion of the movement. At the beginning of the experimental session, at least 10 practice trials were performed in order to check and to adjust the frequency of execution of the movements, as well as to avoid any kind of rhythmicity on the performance and blinking. Special care was taken so that the subjects sat upright during this task, and they were instructed before and during the task about to minimize head movements.

The experimental design consisted in four blocks of movements (10 min per block), each performed with a single arm and two blocks per arm. Arms were alternated (right-left-right-left and vice versa) and the order of alternation was counterbalanced by subjects.

### Hand-tracking system and analysis

A computerized hand-tracking system (CMS-30P, Zebris, Isny, Germany) was used to continuously record the three-dimensional spatial position of an ultrasound marker attached to the metacarpophalangeal joint of the index finger (Figure 1A). Data were sampled at 66 Hz and analyzed with an in-house script using MatLab 7.5 (Mathworks Inc., Natick, MA). The recorded time series of the trajectory of the hand movements were filtered offline using a moving average filter (10 data points) in order to reduce the number of signal artifacts produced by spurious movements during performance. Each data sample consisted in three coordinates (components) that were used for the three-dimensional reconstruction of the trajectory of each movement (Figures 1C and D).

For each component of the time series of the trajectory, we computed off-line the time series of the velocity through numerical differentiation (Hermsdörfer et al., 2003). Typically, in each trajectory, the velocity increased up to a maximum and then decreased again until a local minimum when the target was reached (forward movement). Afterwards, an increase of negative velocity indicated the hand going back movement to the starting position (backward movement).

For each movement, the onset of the forward movement was defined as the first data point which longitudinal component (y-axis, Figure 1D) accomplished three conditions: (i) it should exceed a threshold of velocity of 8 mm/s, (ii) it should be displaced at least 5 mm from the initial position, and (iii) no other in the following 20 points of the time series of velocity (that corresponding approximately to 300 ms) should cross-back the zero-line.

For each movement trajectory and for each hand, several parameters of the performance were considered. Movement time indicated the time invested in reaching the target position. The peak velocity was considered as the maximum value that the velocity achieved during the movement time. As described in Hermsdörfer et al. (2003), the percentage of acceleration time was calculated as the percentage of the whole movement time in which the peak velocity was achieved. The maximum height achieved during the movement time was also determined, as well as the percentage of acceleration time for the height. Finally, the time elapsed between two consecutive movement onsets was calculated.

To test differences between left/right movements performance, paired *t*-test were applied separately for each parameter described above. To analyze the similarity of these parameters between left/right movements, we used the Pearson correlation for each parameter. For the *t*-tests and the Pearson correlations, the significance level was set at *p* = 0.05.

### EEG data acquisition

The EEG signal was recorded continuously (bandpass-filtered 0.01–250 Hz; A/D rate 500 Hz) with a Brainvision system (BrainProducts, Munich, Germany), and analyzed offline using the EEGLAB toolbox (Delorme and Makeig, 2004). An electrode cap was used to record EEG from 29 Ag/AgCl electrodes (Fp1/2, F3/4, C3/4, P3/4, O1/2, F7/8, T3/4, T5/6, Fz, Cz, Pz, FC1/2, FC5/6, CP1/2, CP5/6, PO1/2) using the extended 10–20 system (Jasper, 1958). An external electrode placed on the right ocular canthus was used as reference. The ground electrode was placed on FCz. A VEOG electrode was placed 1 cm below the right eye to detect vertical eye movements, and two additional electrodes were placed on each mastoid, all them recorded against the reference electrode. All impedances were kept below 5 kΩ. Data were bandpass-filtered offline between 0.01 and 45 Hz. Eye-movement artifacts were removed using a second-order blind identification (SOBI) technique (Joyce et al., 2004). EEG data were re-referenced offline to the algebraic summation of both mastoids.

### EEG-tracking system synchronization

The synchronization between the EEG signal and the hand-tracking system was performed to allow the time-to-time correspondence between both time series (ERPs and trajectories). To this end, we used a PC computer with the software Presentation (Neurobehavioral Systems, Albany, CA) that served for simultaneously sending a 5 V electrical squared-wave to both the hand-tracking system and the EEG recorder before each block of movements. We used an in-house-made cable for trigger-out this electric signal through a parallel port and to trigger it in to the tracking system in one side (through a parallel port) and to the EEG recording (through a serial port) in the other side. When the electric squared-wave was received, the continuous recording of the position of the ultrasound sender started. The mark appearing in the EEG recording at this time was later used offline as a synchronization marker between the recordings from the tracking system and the EEG.

### EEG signal analysis

Single-trial EEG data epochs were extracted from the continuous EEG and used for averaging. Epochs were time-locked to the onset of the movement defined using the time series acquired with the hand-tracking system. Each epoch was 7 s long, taking 3 s before and 4 s after the movement onset. The baseline was determined as the average activity in the −2250 to −2000 ms interval preceding each onset. Trials exceeding ±200 μV were rejected. For each participant, at least 100 free-of-artifacts trials were obtained for each arm. The averaged ERPs were transformed into reference-free CSD waveforms using the spherical spline surface Laplacian algorithm (using 4th degree-Legendre polynomials and a smoothing coefficient of 10^−5^) reported by Perrin et al. (1989). The CSD waveforms were computed for each original ERP waveform using a CSD toolbox for MatLab (Kayser and Tenke, 2006).

Time-frequency analysis was performed convolving single-trial data from both ERPs and CSD waveforms with a complex Morlet wavelet (Tallon-Baudry et al., 1997). The frequencies studied ranged from 1 to 40 Hz, with a linear increase of 1 Hz. The time-varying energy was computed for each trial and averaged separately for each subject. The percentage change with respect to a baseline set 2250–2000 ms before the movement onset was extracted and averaged. Percentage of power decrease (ERD) or increase (ERS) of the mu (8–13 Hz) and beta band (17–24 Hz) with respect to baseline were calculated, since these are the most commonly studied in motor tasks (Neuper et al., 2006).

An initial analysis was performed to ensure that the topographic distribution of the ERPs and ERD/ERS were the same for left and right arm movements (See Supplementary material for a further description of the analysis). After demonstrating this, data for left and right arm movements were merged. To maintain the laterality effects, the signal acquired from channels located on the left and right hemisphere were switched for left arm movements. This procedure allowed us to consider all movements as right hand movements.

The statistical analysis was aimed to study specific scalp distributions of the activity during the different phases of the movement based in the velocity time series behavior during the movement performance. We identified the time-windows of interest based on the kinematics of the movement (see definition of these intervals in the Results section). We analyzed differences on the scalp distribution of the activity for each time-window of interest. We divided the set of electrodes into nine different regions: anterior-left (F7, F3, and FC5), anterior-medial (Fz, FC1, and FC2), anterior-right (F4, F8, and FC6), central-left (C3, T3, and CP5), central-medial (Cz, CP1, and CP2), central-right (C4, T4, and CP6), posterior-left (P3, T5, and O1), posterior-medial (Pz, PO1, and PO2) and posterior-right (P4, T6, and O2). First, we conducted an analysis of variance (ANOVA) with factors TIME-COURSE (each time window selected from kinematic data of the movement) × ANTEROPOSTERIOR (anterior regions vs. central regions vs. posterior regions) × LATERALITY (left regions vs. medial regions vs. right regions) to ensure distributional differences between the different times-windows. This clustering was considered in order to reduce the number of degrees of freedom in the statistical analysis (See Figures S1–S3 in Supplementary Material for illustration of the average-waveforms corresponding to each of these clusters for the ERPs, mu and beta power bands). Second, we conducted an ANOVA for each time-course with factors ANTEROPOSTERIOR and LATERALITY, to investigate the distribution of the activity within each time interval. When appropriate, the Greenhouse-Geisser correction was used. In all analyses, the level of significance was set at *p* = 0.05.

## Results

### Behavioral analysis

**Figure 2:**
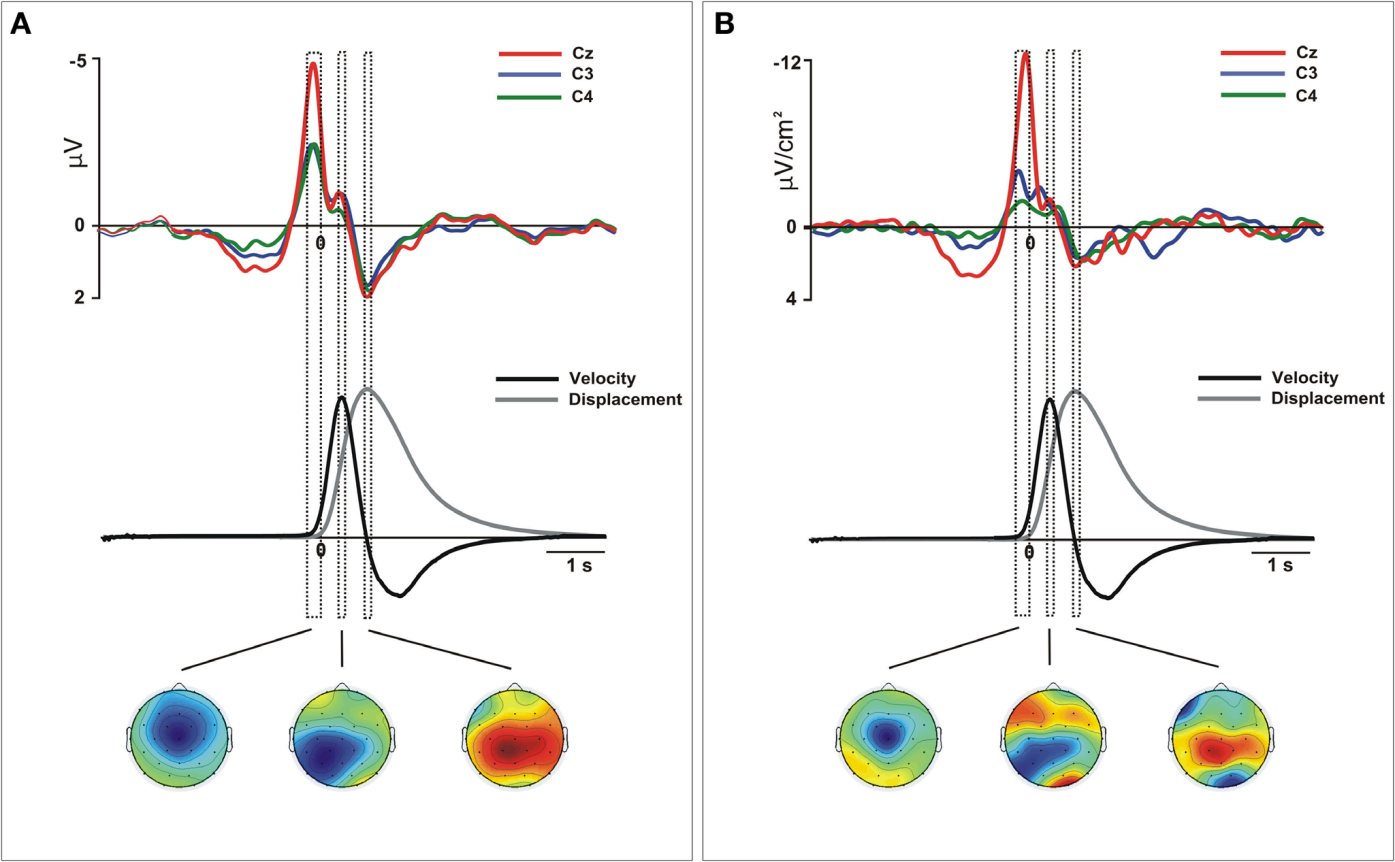
**Movement-related ERPs (A) and their laplacian-transformed CSD waveforms (B) at the locations C3, C4, and Cz time-locked to the onset of the movement for merged left and right movements**. Before merging, electrodes were flipped from one hemisphere to the other for left movements. Below, mean 2-D time series of the trajectory (y-coordinate) of the movement registered with the ultrasound sender (gray). The time series of velocity (black) is represented as the numerical differentiation of the displacement time series. Vertical squares (time-intervals) correspond to different components of the movement-related ERPs that were related with different stages of the movement preparation and execution. Bellow, the topographical distribution of the scalp activity in each of these time-intervals is shown. Warm colors indicate positive activation and cold colors indicate negative activation.

All movements showed a bell-shaped velocity profiles with respect the longitudinal edge. Figure 1C shows the three-dimensional reconstruction of the trajectory of one movement. The recorded kinematic parameters presented the standard characteristics of pointing movements in both arms, that is, single-positive and negative peaks on the velocity and single positive peaks on the displacement (Figure 2, middle).

We did not find differences between right and left movements in movement time [*t*_(14)_ = −0.9, *p* > 0.1], maximum altitude [*t*_(14)_ = 0.5, *p* > 0.1], percentage of acceleration time [*t*_(14)_ = 0.93, *p* > 0.1] and percentage of the acceleration time for the altitude [*t*_(14)_ = 1.46, *p* > 0.1]. Only a slight but non-significant difference in maximum velocity [*t*_(14)_ = −1.9, *p* = 0.08] was found, being left movements slightly faster than right movements. Furthermore, the number of movements performed for both hands was similar [*t*_(14)_ = 0.58, *p* > 0.1]. Indeed, the elapsed time between two consecutive forward movement onsets (mean ± SD) was 8.4 s ± 2.7 for the right arm movements and 8.6 s ± 2.3 for the left arm movements. Movement time (*r* = 0.96, *p* < 0.01), maximum velocity (*r* = 0.73, *p* < 0.01), maximum altitude (*r* = 0.62, *p* < 0.01) and the latency of the peak of maximum velocity relative of the movement time (*r* = 0.85, *p* < 0.01) were all strongly correlated for left and right movements. By contrast, the latency of the peak of the maximum altitude relative to the movement time did not show a significant correlation between hands (*r* = 0.4, *p* > 0.1). For further details about the behavioral performance, see Table S1 in Supplementary material.

### Electrophysiological data

The statistical analysis did not reveal differences on the distribution as a function of the active hand and we merged the epochs corresponding to the neural activity obtained during left and right arm movements (See Supplementary material for details of this analysis). Figure 2 shows the waveforms extracted from the grand mean ERPs and CSD over the contralateral, ipsilateral and medial motor regions (see Figure S4 for details of data from the right and left hand separately). Single-trial epochs were averaged time-locked to the movement onset detected using the time series of velocity. For both ERPs and CSD-transformed epochs, we performed an analysis to ensure that the amplitudes did not change between left (non-dominant) and right hand (dominant) movements during preparation and execution phases. To this aim, we divided the interval from 1 s before to 1 s after the movement onset in sub-intervals of 100 ms each. This selection included the EEG signal corresponded to the preparation and execution of the movement. We included into the ANOVA the mean amplitude of the activity over the selected regions in each time interval for both arm movements. After, we proceed to merge the epochs obtained from left and right arm movements. As it is shown in Figure 2, we selected three time-intervals for the statistical analysis. This selection was based on the kinematics of the movement: from −200 to 0 ms (prior to the movement onset), from 250 to 350 ms (time-interval corresponding to the peak velocity) and from 700 to 800 ms after the onset (corresponding with the offset of the forward movement).

#### ERPs grand mean

**Figure 3:**
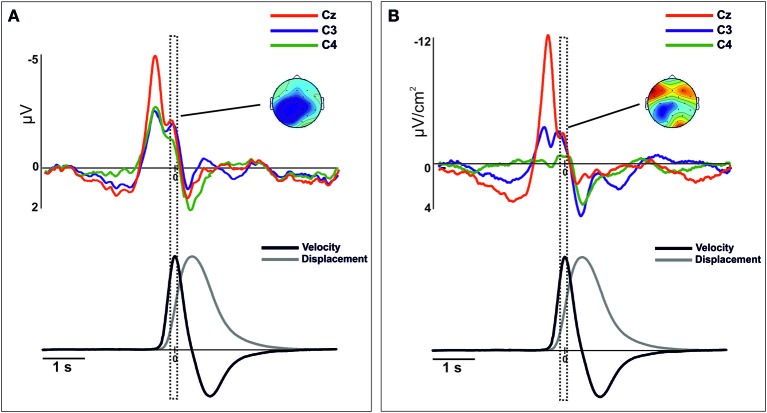
**Movement-related ERPs (A) and their laplacian-transformed CSD waveforms (B) at the locations C3, C4, and Cz time-locked to the peak-velocity**. Before merging, electrodes were flipped from one hemisphere to the other for left movements. Below, mean 2-D time series of the trajectory (y-coordinate) of the movement registered with the ultrasound sender (gray). The time series of velocity (black) is represented as the numerical differentiation of the displacement time series. Vertical square include the time-interval (centered at the 0) that corresponds to the topographic representation. Warm colors indicate positive activation and cold colors indicate negative activation. To note, there is a clear degree of similarity between the component (−50 to 50 ms) and its distribution than the observed in Figure 2.

After merging epochs from both arms, we found a significant effect of TIME-COURSE [*F*_(2, 28)_ = 16.9, *p* <0.01], and a significant interaction TIME COURSE × ANTEROPOSTERIOR × LATERALITY [*F*_(8, 112)_ = 16.79, *p* < 0.01] indicating changes of the scalp distribution of the activity during the performance of movements. Figure 2A shows the ERPs waveforms at the scalp locations C3, C4, and Cz. In this figure we show the concomitant temporal evolution of these waveforms and the time series of displacement and velocity of the marker. First, we observed a big negative deviation starting prior to the movement onset that achieved its maximum at −100 ms. This activity showed a clear central and anterior-medial distribution [time-interval −200 to 0 ms, ANTEROPOSTERIOR × LATERALITY, *F*_(4, 56)_ = 21.67, *p* < 0.01] (Figure 2A, below). Additionally, in order to seek for a significant lag between the peak activity corresponding to the contralateral M1 and the SMA, we conducted a paired *t*-test between the latencies of both peaks. We did not find a significant time-lag between these two peak activity [*t*_(14)_ = 0.27, *p* > 0.5]. At the time to peak velocity, the distribution of the activity became mainly posterior and contralateral to the movement side [time-interval 250 to 350 ms, ANTEROPOSTERIOR × LATERALITY, *F*_(4, 56)_ = 5.62, ε = 0.61, *p* < 0.01]. In order to corroborate this result, we calculated again the epochs of the ERPs locked trial-by-trial to the peak velocity. Figure 3A shows the ERP-waveforms at the scalp locations C3, C4, and Cz. Again, we observed this negative component that was maximal at 0 ms coincident to the peak velocity and showing the same distribution through the scalp [time-interval −50 to 50 ms, ANTEROPOSTERIOR × LATERALITY *F*_(4, 56)_= 13.49, ε = 0.57, *p* < 0.01]. Finally, we found a slow positive deviation starting at 350 ms after the movement onset, peaking at the end of the forward movement. In this time-interval, the distribution of the activity was anterior-medial and bilateral, greater in regions ipsilateral to the movement [time-interval 700–800 ms, ANTEROPOSTERIOR × LATERALITY, *F*_(4, 56)_ = 11.18, *p* < 0.01].

#### CSD grand mean

After merging data, we found differences of distribution of the CSD-transformed activity over the whole scalp between these three time-courses [TIME-COURSE × ANTEROPOSTERIOR × LATERALITY, *F*_(4, 56)_ = 4.65, *p* < 0.01]. A first negative component was found starting 1000 ms before the movement onset, being maximal around −100 ms. This activity was prominently distributed in centro-medial regions [time-interval −200 to 0 ms, ANTEROPOSTERIOR × LATERALITY, *F*_(4, 56)_ = 32.45, *p* < 0.01], as it is shown in Figure 2B. Similarly as we did for the ERPs grand mean analysis, we sought for significant differences in the peak latencies between contralateral M1 and the SMA. Again, we did not find a significant time-lag between these activities [*t*_(14)_ = 0.58, *p* > 0.5]. Coincident to the peak velocity, we found a sink distributed over the posterior region, contralateral to the movement side. In addition, a current source distributed in frontal regions was observed at this time, more prominent over the contralateral regions [time-interval 250–350 ms, ANTEROPOSTERIOR × LATERALITY, *F*_(4, 56)_ = 11.64, *p* < 0.01]. When locking the epochs trial-by-trial to the peak velocity, we found similar CSD-waveforms at the locations C3, C4, and Cz as observed locking the activity to the movement onset, as well as the scalp distribution [ANTEROPOSTERIOR × LATERALITY, *F*_(4, 56)_ = 11.9, *p* < 0.01] (see Figure 3B). Finally, at the end of the forward movement, we found a source distributed in post-central bilateral regions, more prominent over regions contralateral to the side of the movement [time-interval 700–800 ms, ANTEROPOSTERIOR × LATERALITY, *F*_(4, 56)_ = 14.6, *p* < 0.01].

### Time frequency-analysis

**Figure 4:**
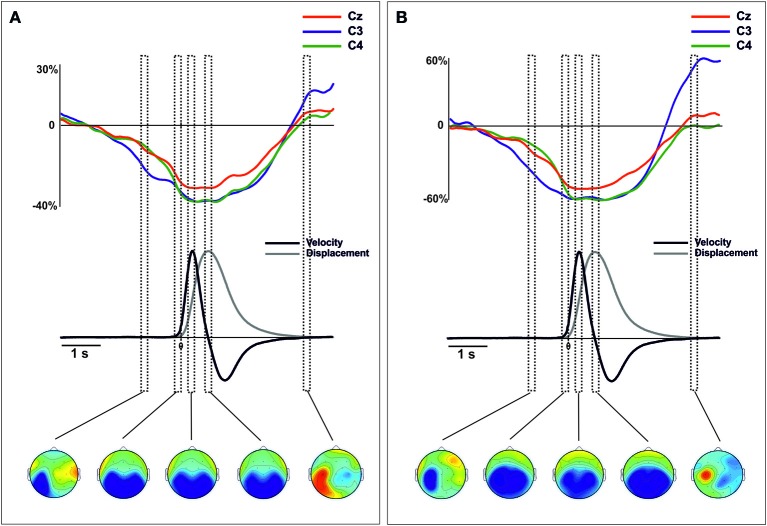
**Grand average traces of mu (8–13 Hz) ERD/ERS extracted from voltage (A) and CSD-transformed signal (B) for electrodes C3, C4, and Cz time-locked to the onset of the movement**. Values are in percentages of the base-line period (−2250 to −2000 ms). Before merging, electrodes were flipped from one hemisphere to the other for left movements. Below, mean 2-D time series of the trajectory (y-coordinate) of the movement registered with the ultrasound sender (gray). The time series of velocity (black) is represented as the numerical differentiation of the displacement time series. Vertical squares (time-intervals) correspond to different components of the ERD/S that were related with different stages of the movement preparation and execution. Bellow, the topographical distribution of the power synchronization and desynchronization is shown. Warm colors indicate increases of synchronization and cold colors indicate increases of desynchronization.

**Figure 5:**
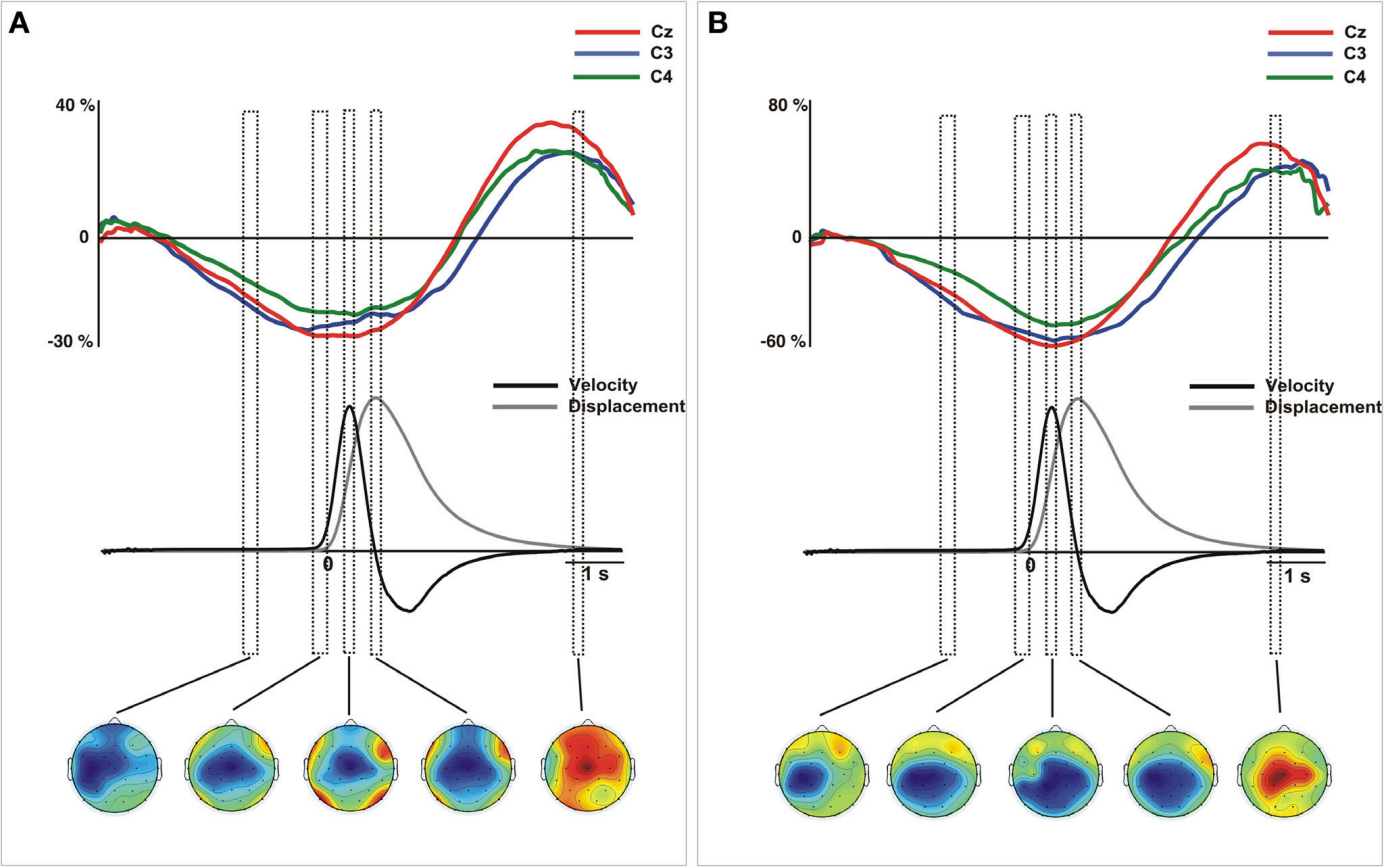
**Grand average traces of beta (18–24 Hz) ERD/ERS extracted from voltage (A) and CSD-transformed signal (B) for electrodes C3, C4, and Cz time-locked to the onset of the movement**. Values are in percentages of the base-line period (−2250 to −2000 ms). Before merging, electrodes were flipped from one hemisphere to the other for left movements. Below, mean 2-D time series of the trajectory (y-coordinate) of the movement registered with the ultrasound sender (gray). The time series of velocity (black) is represented as the numerical differentiation of the displacement time series. Vertical squares (time-intervals) correspond to different components of the ERD/S that were related with different stages of the movement preparation and execution. Bellow, the topographical distribution of the power synchronization and desynchronization is shown. Warm colors indicate increases of synchronization and cold colors indicate increases of desynchronization.

Similarly to the ERPs and CSD waveforms, we merged the epochs corresponding to the oscillatory brain activity obtained from left and right arm movements (See Supplementary material for the description of this procedure). Figures 4, 5 show the mu- and beta-ERD/S at locations C3, C4, and Cz locked to the movement onset (see Figures S5, S6 for details of data from the right and left hand separately). In both power-bands we found a large desynchronization over the contralateral, ipsilateral and central motor areas, starting around 1500 ms before the movement onset, lasting until 2000 s after the movement onset. In addition, a post-movement synchronization in both power bands was found starting 2300 ms after the movement onset. This synchronization was extended in regions contralateral to the side of the movement, more prominently in the mu-band (see Figure 4).

In addition to the intervals considered in the statistical analysis of the ERPs, we included two other intervals corresponding to the early preparation of the movement (−1000 to −800 ms) and the end of the whole movement (3200–3400 ms).

#### Time-frequency derived from ERPs

The analysis of the mu-band extracted from the merged data indicated different and specific spatial distributions in each time-interval [TIME-COURSE × ANTEROPOSTERIOR × LATERALITY *F*_(6, 84)_ = 4.14, *p* < 0.01]. During the preparation of the movement, the mu-ERD showed a clear distribution over posterior regions contralateral to the movement side [time-interval −1000 to −800 ms, ANTEROPOSTERIOR × LATERALITY, *F*_(4, 56)_ = 3.97, *p* < 0.01] (Figure 4A). From the onset of the movement until reaching the target location, the mu-ERD was distributed mainly over posterior and bilateral regions [time intervals from −200 to 0 ms, from −250 to 350 and 700 to 800 ms, ANTEROPOSTERIOR, *F*_(2, 28)_ > 17.7, *p* < 0.01 in all intervals]. At the end of the whole movement, a clear mu-ERS arose in regions contralateral to the movement side [time-interval 3200–3400 ms, ANTEROPOSTERIOR × LATERALITY, *F*_(4, 56)_ = 3.58, *p* < 0.05].

The power mu-band extracted from the CSD transformed data also showed specific spatial distributions in each time-interval [TIME-COURSE × ANTEROPOSTERIOR × LATERALITY, *F*_(6, 84)_ = 5.005, *p* < 0.01]. The mu-ERD obtained from these data showed a distribution over the posterior contralateral regions, similarly to that obtained from voltage signal [time-interval −1000 to −800 ms, ANTEROPOSTERIOR × LATERALITY, *F*_(4, 56)_ = 2.86, *p* < 0.05]. Differently, however, the topographical distribution revealed a more enclosed activity within these regions that the observed from voltage data (Figure 4B, bottom). Again, no differences on the distribution of the mu-ERD were found in time intervals covering the onset, the peak velocity and the end of the forward movement [time intervals from −200 to 0 ms, from −250 to 350 and 700 to 800 ms, ANTEROPOSTERIOR, *F*_(2, 28)_ > 6.07, *p* < 0.01 in all intervals]. Similarly to the mu-band power obtained from the voltage signal, we found a clear ERS over central and contralateral to the movement side [time-interval 3200–3400 ms, ANTEROPOSTERIOR × LATERALITY *F*_(4, 56)_ = 2.78, *p* < 0.05], more localized over contralateral motor regions (see Figure 4B, bottom). For the beta band, we did not find a significant effect of TIME-COURSE [*F*_(3, 42)_ = 0.52, *p* > 0.1] in the merged data. However, we found significant interactions of TIME-COURSE × LATERALITY [*F*_(6, 84)_ = 3.16, ε = 0.61, *p* < 0.05] and TIME-COURSE × ANTEROPOSTERIOR [*F*_(6, 84)_ = 2.41, *p* < 0.05 ] suggesting certain specificity of the distribution of the oscillatory beta power activity as a function of the time-intervals. During the preparation of the movement, the beta-ERD was distributed prominently over central and contralateral regions (see Figure 5A) [time-interval −1000 to −800 ms, ANTEROPOSTERIOR × LATERALITY, *F*_(4, 56)_ = 2.74, *p* < 0.05] that became larger at bilateral and medio-central regions during the onset of the movement [time-interval −200 to 0 ms, ANTEROPOSTERIOR × LATERALITY, *F*_(4, 56)_ = 3.07, *p* < 0.05]. At the peak velocity, we did not find a clear distribution of the ERD [time-interval 250–350 ms, ANTEROPOSTERIOR × LATERALITY, *F*_(4, 56)_ = 1.93, *p* > 0.1]. However, data showed a large beta-ERD over central and medial regions [time-interval 250–350 ms, ANTEROPOSTERIOR, *F*_(2, 28)_ = 4.2, *p* < 0.05; LATERALITY, *F*_(2, 28)_ = 6.67, *p* < 0.05]. We found a larger desynchronization over centromedial regions at the end of the forward movement [time-interval 700–800 ms, ANTEROPOSTERIOR × LATERALITY, *F*_(4, 56)_ = 2.99, ε = 0.609, *p* < 0.05]. Finally, we found an increase of power synchronization (ERS) starting 2 s after the movement with a clear distribution over centromedial regions [time-interval 3200–3300 ms, ANTEROPOSTERIOR × LATERALITY, *F*_(4, 56)_ = 3.1, *p* < 0.05].

#### Time frequency derived from CSD-transformed data

The power mu-band extracted from the CSD transformed data also showed specific spatial distributions in each time-interval [TIME-COURSE × ANTEROPOSTERIOR × LATERALITY, *F*_(6, 84)_ = 5.05, *p* < 0.01]. The mu-ERD obtained from these data was distributed over the posterior contralateral regions, similarly to that obtained from voltage signal [time-interval −1000 to −800 ms, ANTEROPOSTERIOR × LATERALITY, *F*_(4, 56)_ = 2.86, *p* < 0.05] (Figure 4B, bottom). Again, no differences on the distribution of the mu-ERD were found in time intervals covering the onset, the peak velocity and the end of the forward movement [time intervals from −200 to 0 ms, from −250 to 350 and 700 to 800 ms, ANTEROPOSTERIOR, *F*_(2, 28)_ > 6.07, *p* < 0.01 in all intervals]. Similarly to the mu-band power obtained from the voltage signal, we found a clear ERS over central and contralateral to the movement side [time-interval 3200–3400 ms, ANTEROPOSTERIOR × LATERALITY *F*_(4, 56)_ = 2.78, *p* < 0.05], more localized over contralateral motor regions (see Figure 4B, bottom).

Regarding the oscillatory activity within the beta band from the CSD transformed signal, we again found certain similarity with that obtained from the voltage signal. After merging, we found a significant effect of TIME-COURSE [*F*_(3, 42)_ = 9.61, *p* < 0.01], and also significant interaction of TIME-COURSE × ANTEROPOSTERIOR [*F*_(6, 84)_ = 4.28, ε = 0.38, *p* < 0.05] and TIME-COURSE × LATERALITY [*F*_(6, 84)_ = 3.655, ε = 0.331, *p* < 0.05]. During the preparation of the movement, beta-ERD was mainly distributed over central contralateral regions [time-interval −1000 to 800 ms, ANTEROPOSTERIOR × LATERALITY, *F*_(4, 56)_ = 5.63, *p* < 0.01] (see Figure 5B). During the onset of the movement, the distribution of the ERD shifted toward mediocentral regions and contralateral regions [time-interval −200 to 0 ms, ANTEROPOSTERIOR × LATERALITY, *F*_(4, 56)_ = 3.4, *p* < 0.05], and remained similar during the execution until the end of the forward movement. At the end of the movement, a beta-ERS was found, showing a clear distribution over contralateral-central regions [time-interval 3200–3400 ms, ANTEROPOSTERIOR × LATERALITY, *F*_(4, 56)_ = 2.87, *p* < 0.05].

## Discussion

The main purpose of the present study was to investigate the association between the fluctuation of the time series of velocity during the performance of multi-joint reaching movements and (i) the components of the MRBPs and (ii) the ERD/S in the mu and beta-bands. We found three amplitude-peaks in the components of the MRBPs corresponding to specific time intervals within the preparation and execution phases of movements. These components have been clearly associated with the dynamics of the time series of velocity obtained from the trajectories of the movements recorded with a hand-tracking system. We show a novel approach to investigate the components of the movement-related brain activity brain activity of multi-joint self-paced movements as a function of the changes of the velocity pattern during their performance.

### Behavioral data

The analysis of movement trajectories showed the standard characteristics of pointing movements in both arms (Kirsch et al., 2010). With regard to the time series of velocity, a first positive peak after the movement onset indicated the peak velocity during the forward movement. Following, a negative peak corresponded to the peak velocity during the backward movement. Interestingly, behavioral parameters showed a high degree of similarity between right (dominant) and left (non-dominant) hand movement. This seems to be contradictory as the performance of both hands should differ as a consequence of the function lateralization (Lavrysen et al., 2012). There are a considerable number of studies suggesting that right arm advantages (in right-handers) might exist for kinematic parameters such as movement velocity and movement time (Hoffmann, 1994; Elliott et al., 1995; Sainburg and Kalakanis, 2000). Sainburg and Kalakanis (2000) found differences in the magnitude of the left/right shoulder muscle torque during reaching movements, indicating that control of both limbs might be underlined by different neural sources. Nevertheless, more recent studies support the idea that differences between the dominant and non-dominant sides arise in other aspects of motor performance than purely kinematics (Sainburg, 2002; Wang and Sainburg, 2007), such as the strength at the initiation of the movement or the selected strategy to achieve the target. Our findings seem to point in this direction, given that our measurements explain kinematic characteristics of the movement rather than other qualitative parameters (e.g., the median deviation of movement path) which are dependent of the handedness of the subject. Nonetheless, the acquisition of the time series of position at more locations of the arm, such as the shoulder of the fore-arm, would definitively allow a more fine-grained comparison of kinematic properties of left/right movements. Therefore, we should remain speculative about this concern.

### Electrophysiological recordings

Most of the studies of MRBPs have used the EMG activity acquired with attached skin electrodes to identify the movement onset in absence of an external trigger (Deecke et al., 1969, 1980; Berardelli et al., 1996; Mackinnon and Rothwell, 2000). In our paradigm we used the signal recorded from the ultrasound marker to determine the movement onset using a velocity threshold. The use of ultrasonic signal to categorize trials in function of the velocity has been previously used to study the sensitivity of evoked brain activity to the range of motion in rapid goal-directed movements (Kirsch and Hennighausen, 2010; Kirsch et al., 2010), as well as synchronize the EEG signal to the movement onset for extracting the epochs (Bradberry et al., 2010). However, this is the first time that both time series (trajectory-based and EEG) are analyzed together with the aim to find associations between the characteristics of the MRBPs-components and the kinematics during the performance of natural movements.

The MRBPs during the multi-joint outback movements showed, for both voltage and CSD waveforms, a series of deflections that have been reported previously as accompanying ballistic movements (Berardelli et al., 1996; Babiloni et al., 1999; Cui et al., 1999). As a novelty, we establish a point to point association between the time series of velocity of movements and specific components of the MRBPs during movement performance. First, we observe a negative component that peaks at few tens of milliseconds prior to the onset that might correspond to the late-BP (Shibasaki and Hallett, 2006). Previous studies have reported the recruitment of the supplementary motor area (SMA) during the preparation of the movement, as well as the involvement of the contralateral primary motor regions immediately before the onset using different techniques such as EEG (Deecke et al., 1980; Cui et al., 1999; Ohara et al., 2006), event-related functional magnetic resonance imaging (Cunnington et al., 2003, 2005) and magnetoencephalography (MEG) (Cheyne et al., 1991; Nagamine et al., 1996; Erdler et al., 2000). As expected from these studies, our data reveal a prominent activation of fronto-central regions during the late-BP which would indicate the recruitment of the SMA. Another possible explanation for the increase of the activity in the SMA is associated to the role of this area in time estimation. Indeed, in this task we asked participants to wait a concrete period of time (7–10 s) between two consecutive movements. Several studies pointed the role of SMA in the attentional modulation of the time estimation (Coull et al., 2004; Schwartze et al., 2012). This possible explanation could not be ruled-out in this study. However, the SMA activation that we observed is very similar in distribution and latency to that observed in other studies using the same kind of paradigms involving self-paced motor programs (see Shibasaki and Hallett, 2006, for review) which is considered to be motor-related. To note, we found that the SMA activity did not precede the activity of the contralateral M1 as would be expected taking into account the hierarchical organization of the motor system, as suggested in previous studies (Vidal et al., 2003). This time-lag between these activities has been clearly observed in tasks involving response-choice (see Carbonnell et al., 2004). However, the task here described consists in the repetition of movements that are identically performed along the whole task, which might reduce the hierarchical flow of activity within the motor system. This could explain the apparent coincidence in time of these peaks of activity corresponding with these two structures.

A second selected stage of the movement corresponds to the peak velocity. In this period, a second negative component was observed, being maximal over parietal areas contralateral to the movement onset. We confirmed this finding epoching the MRBPs to this time point, which showed a similar behavior. Therefore, it seems that this activation might indicate a neural substrate of the encoding of the kinematics of movements. Such activity is in agreement with previous studies that reported increments of activity over the posterior parietal cortex (PPC) in sensorimotor processes during visuomotor reaching movements (Reichenbach et al., 2014). However, to our knowledge, we report by first time a clear relationship between this neural activity and the kinematics of the movement, concretely the achievement of the peak velocity. This result shows the active role of the PPC, not only in encoding the afferent input from the sensory system, but also in other processes related with monitoring the kinematics of the movement. In a very brilliant study, Bourguignon et al. (2012) reported evidences from MEG about the pivotal role of the left posterior parietal cortex in the integration of sensorimotor features of limb kinematics, which might agree with the enrolment of this area in processing velocity changes during movement performance.

Finally, a large positive activation arises on both motor cortices when the target is achieved, mainly distributed over contralateral parieto-central regions. Few studies have provided evidence of changes in corticospinal excitability accompanying voluntary relaxation of a muscle. Transcranial magnetic stimulation studies have reported a decreased motor evoked potentials (MEP) in the contracting muscle related with the decrease of the EMG signal from the same muscle at the offset of movements (Waldvogel et al., 2000). In addition, positive motor-related movement potential has been defined as an inhibitory process, which is in agreement with our findings.

### Event-related synchronization/desynchronization

In addition to ERPs, we investigated whether the activity and the scalp distribution of the ERD/S in the mu and beta bands were also related with kinematic properties of the movement. During the preparation and the execution of movements, we found the same pattern of synchronization and desynchronization in both bands as reported previously (Pfurtscheller and Aranibar, 1977, 1979; Pfurtscheller et al., 1996; Stancák Jr and Pfurtscheller, 1997; Alegre et al., 2003, 2004a,b). This oscillatory activity has been largely considered an indicator of neural activation during motor tasks (Salmelin et al., 1995). However, we did not find any specific distribution of this ERD associated with to the kinematics of the movement as we found with the MRBPs. It has been suggested that the sources of MRBPs and those related to the oscillatory brain activity may have different roles during movement execution. In such case, our findings would support this hypothesis. Of interest, we found a certain overlapping in these results when we studied the oscillatory brain activity of the voltage and the CSD. Notably, however, the CSD maps evidenced a superior performance localizing the scalp regions with the maximal activation in both mu and beta power activity. This is consequence of the Laplacian transformation appliying a spatial high-pass filtering, which avoids the contribution of spurious remote activities in calculating the sources (Tenke and Kayser, 2012).

### Clinical applications

An important aspect of this study is the use of a hand-tracker to extract the kinematic aspects of movement performance. This method establishes a potential tool to study the evolution of the EEG related to the intrinsic properties of the movement performance. In our view, this approach could be useful in clinical studies. Indeed, hand-trackers are used to evaluate several clinical scores of the quality of movement in patients suffering from stroke consequences (Hermsdörfer and Goldenberg, 2002) and focal dystonia (Berardelli et al., 1996; Ruiz et al., 2011). We believe that the application of this experimental setup would help to disentangle specific patterns of brain activity associated to the behavioral outcome of movements. Furthermore, longitudinal studies could also benefit from this method, allowing the study of changes in brain activity and performance due rehabilitative interventions (Amengual et al., 2012; Grau-Sánchez et al., 2013).

Particularly, EMG is very sensitive to spurious activity that records from skin and it is inevitably contaminated by artifacts especially in clinical studies (Olier et al., 2011). This undesirable activity may alter the interpretation of the EMG signal when relating muscular activation to movements (De Luca et al., 2010; Olier et al., 2011). In addition, besides the valuable physiological information that EMG signal provides, specific kinematic properties of the movement are missed. Instead, signal recorded from ultrasonic markers allows a better understanding of the kinematics of movement and an easier detection of movement changes than using EMG. However, more cross-modal studies are needed to compare and validate both signals.

### Limitations of this study

This study shows a set of limitations that will be discussed in this section. First, we only used a single marker of the hand-tracking system to register the position of the hand during the performance of movements. Although this montage was fair enough to extract the time series of the velocity and to identify associations between brain activity and kinematics, more markers attached to different locations on the arm, such as the shoulder and the forearm, would provide finer information about the dynamics of joint-muscles during movements and their relation with the EEG activity (Wang and Sainburg, 2007). This additional information could be helpful to confirm the left/right similarities that we found in the kinematic parameters included in our behavioral analysis. A second caveat of this study is the limited temporal resolution of the hand-tracking system (66 Hz) compared to the sampling rate of the EEG signal (250 Hz). Such difference in the frequency of acquisition of these signals might result in a reduced accuracy when locking the MRBPs to the response onset compared to more standardized methods using the EMG signal. However, providing an improved method for locking EEG to the movement onset than those EMG-based was beyond the scope of our study. Instead, we aimed to propose this method as a different manner to look at these motor potentials, as they allow direct comparison between the changes of brain electrical activity and the kinematics during movement execution, which has not been previously described. Indeed, to rule out that such inaccuracy locking the EEG to the motion signal might have caused a significant variability in our data that could explain the component found at the time period of the peak velocity, we extracted the ERPs locked to this time point in a subsequent analysis obtaining exactly the same pattern of activity. Therefore, this method seems reliable enough to study the motor related brain activity associated with the kinematics of movement performance. Future studies should address this issue including the recording of the EMG activity in the same experimental setup reported here. This would allow to compare the ERPs extracted by both locking methods (EMG and hand-tracking system), as well as obtaining a quantitative value of the inaccuracy acquired with the method that we report. A third limitation of this study is the reduced number of electrode locations for the EEG recordings, which may represent an impact for an optimal estimation of the Laplacian transformation of the EEG signal (Yao and Dewald, 2005). However, CSD waveforms extracted with similar algorithms based in spline interpolation have been previously used with the same number of electrodes or even fewer (Carbonnell et al., 2004; Tandonnet et al., 2005; Meckler et al., 2010). Another limitation regarding the application of the Laplacian transformation of the EEG signal concerns to the mean inter-electrode distance of our montage (~5 cm). Early reports suggest that the accuracy of cortical source localization methods decreases as a function of the distance between electrodes considered in the model (Law et al., 1993). However, Giard et al. (2013) suggest that the optimal number of electrodes would range between 30 and 50 in order to avoid errors between the theroretical and real electrode position (that is sensitive to the number of electrodes). In this sense, our montage consists in 29 electrode positions, barely below the threshold defined Giard et al. (2013). Yet, CSD-waveforms are considered a sound method to extract the neural sources in sensorimotor tasks (Tenke and Kayser, 2012), and a low number of electrodes might not affect the reliability of our findings (Ohara et al., 2006).

### Conflict of interest statement

The authors declare that the research was conducted in the absence of any commercial or financial relationships that could be construed as a potential conflict of interest.

## Supplementary material

Figure S1 **ERPs (A) and CSD-transformed waveforms (B) at the clustered locations over the scalp, time-locked to the onset of the movement**. The activity corresponding to the merged left and right movements is shown. Before merging, regions were flipped from one hemisphere to the other for left movements. FL, frontal left; FM, frontal medial; FR, frontal right; CL, central left; CM, central medial; CR, central right; PL, posterior left; PM, posterior medial; PR, posterior right.

Figure S2 **Mu-ERD/ERS (8–13 Hz) extracted from voltage (A) and CSD-transformed waveforms (B) at the clustered locations over the scalp, time-locked to the onset of the movement**. The activity corresponding to the merged left and right movements is shown. Before merging, regions were flipped from one hemisphere to the other for left movements. FL, frontal left; FM, frontal medial; FR, frontal right; CL, central left; CM, central medial; CR, central right; PL, posterior left; PM, posterior medial; PR, posterior right.

Figure S3 **Beta-ERD/ERS (18–24 Hz) extracted from voltage (A) and CSD-transformed waveforms (B) at the clustered locations over the scalp, time-locked to the onset of the movement**. The activity corresponding to the merged left and right movements is shown. Before merging, regions were flipped from one hemisphere to the other for left movements. FL, frontal left; FM, frontal medial; FR, frontal right; CL, central left; CM, central medial; CR, central right; PL, posterior left; PM, posterior medial; PR, posterior right.

Figure S4 **Movement-related ERPs (A) and their laplacian-transformed CSD waveforms (B) at the locations C3, C4, and Cz time-locked to the onset of the movement separately for left and right movements**. Below, mean 2-D time series of the trajectory (y-coordinate) of the movement registered with the ultrasound sender (gray). The time series of velocity (black) is represented as the numerical differentiation of the displacement time series. Vertical squares (time-intervals) correspond to different components of the movement-related ERPs that were related with different stages of the movement preparation and execution. Bellow, the topographical distribution of the scalp activity in each of these time-intervals is shown. Warm colors indicate positive activation and cold colors indicate negative activation.

Figure S5 **Grand average traces of mu (8–13 Hz) ERD/ERS extracted from voltage (A) and CSD-transformed signal (B) for electrodes C3, C4, and Cz time-locked to the onset of the movement separately for left and right movements**. Values are in percentages of the base-line period (−2250 to −2000 ms). Below, mean 2-D time series of the trajectory (y-coordinate) of the movement registered with the ultrasound sender (gray). The time series of velocity (black) is represented as the numerical differentiation of the displacement time series. Vertical squares (time-intervals) correspond to different components of the ERD/S that were related with different stages of the movement preparation and execution. Bellow, the topographical distribution of the power synchronization and desynchronization is shown. Warm colors indicate increases of synchronization and cold colors indicate increases of desynchronization.

Figure S6 **Grand average traces of beta (18–24 Hz) ERD/ERS extracted from voltage (A) and CSD-transformed signal (B) for electrodes C3, C4, and Cz time-locked to the onset of the movement separately for left and right movements**. Values are in percentages of the base-line period (−2250 to −2000 ms). Below, mean 2-D time series of the trajectory (y-coordinate) of the movement registered with the ultrasound sender (gray). The time series of velocity (black) is represented as the numerical differentiation of the displacement time series. Vertical squares (time-intervals) correspond to different components of the ERD/S that were related with different stages of the movement preparation and execution. Bellow, the topographical distribution of the power synchronization and desynchronization is shown. Warm colors indicate increases of synchronization and cold colors indicate increases of desynchronization.
