# Supplementary material for: Linking motor-related brain potentials and velocity profiles in multi-joint arm reaching movements
Source: Front Hum Neurosci. 2014 Apr 29;8:271. doi: 10.3389/fnhum.2014.00271 (PMC4010756; doi:10.3389/fnhum.2014.00271)
Supplement: Figure S1 — ERPs (A) and CSD-transformed waveforms (B) at the clustered locations over the scalp, time-locked to the onset of the movement. The activity corresponding to the merged left and right movements is shown. Before merging, regions were flipped from one hemisphere to the other for left movements. FL, frontal left; FM, frontal medial; FR, frontal right; CL, central left; CM, central medial; CR, central right; PL, posterior left; PM, posterior medial; PR, posterior right. [file DataSheet1.DOCX]

**Supplementary material**

In this section we show the statistical analysis performed to justify the merging data from right and left hand movements.

For the ERPs and the ERD/ERDs, we divided the time window from 1000 ms before until 1000 ms after the movement onset in segments of 100 ms. We introduced the mean values of each dependent variable from each of the nine regions, and we performed a repeated measures ANOVA with factors SIDE (left arm movement vs. right arm movement), TIME (time segments of 100 ms each, further definition given in results) ANTEROPOSTERIOR (frontal regions vs. central regions vs. posterior regions) and LATERALITY (ipsilateral regions vs. medial regions vs. contralateral regions).

We did not find differences between right and left arm movements on the distribution of the brain activity over the whole scalp during the preparation and execution of movements, neither in voltage [SIDE x TIME x ANTEROPOSTERIOR x LATERALITY, *F*(56, 784) = .89, *p* = .68] nor CSD activity [SIDE x TIME x ANTEROPOSTERIOR x LATERALITY, *F*(56,784) = 0.94, *p* = .61] (figure S4).

We proceeded identically for the ERD/S within the mu- and beta- power bands. We did not find differences in the distribution of the beta-power as a function of the active hand, neither in voltage [SIDE x TIME x ANTEROPOSTERIOR x LATERALITY, *F*(56,784) = 1,11, *p* >.1] nor in CSD [TIME x SIDE x ANTEROPOSTERIOR x LATERALITY, *F*(56,784) = 0.94, *p* > .1] (Figure S5).

We obtained the same results for the beta power band for voltage [SIDE x TIME x ANTEROPOSTERIOR x LATERALITY, *F*(56,784) = 0.96, *p* = .61] and CSD activity [TIME x SIDE x ANTEROPOSTERIOR x LATERALITY, *F*(56,784) = 0.81, *p* = .968] (figure S6).


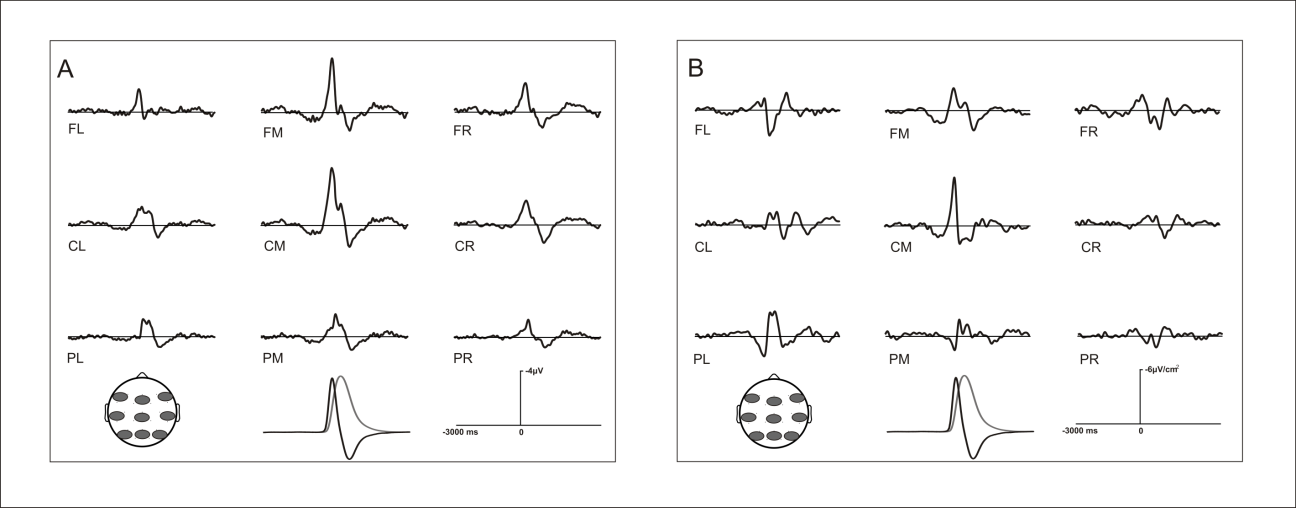


**Figure S1**


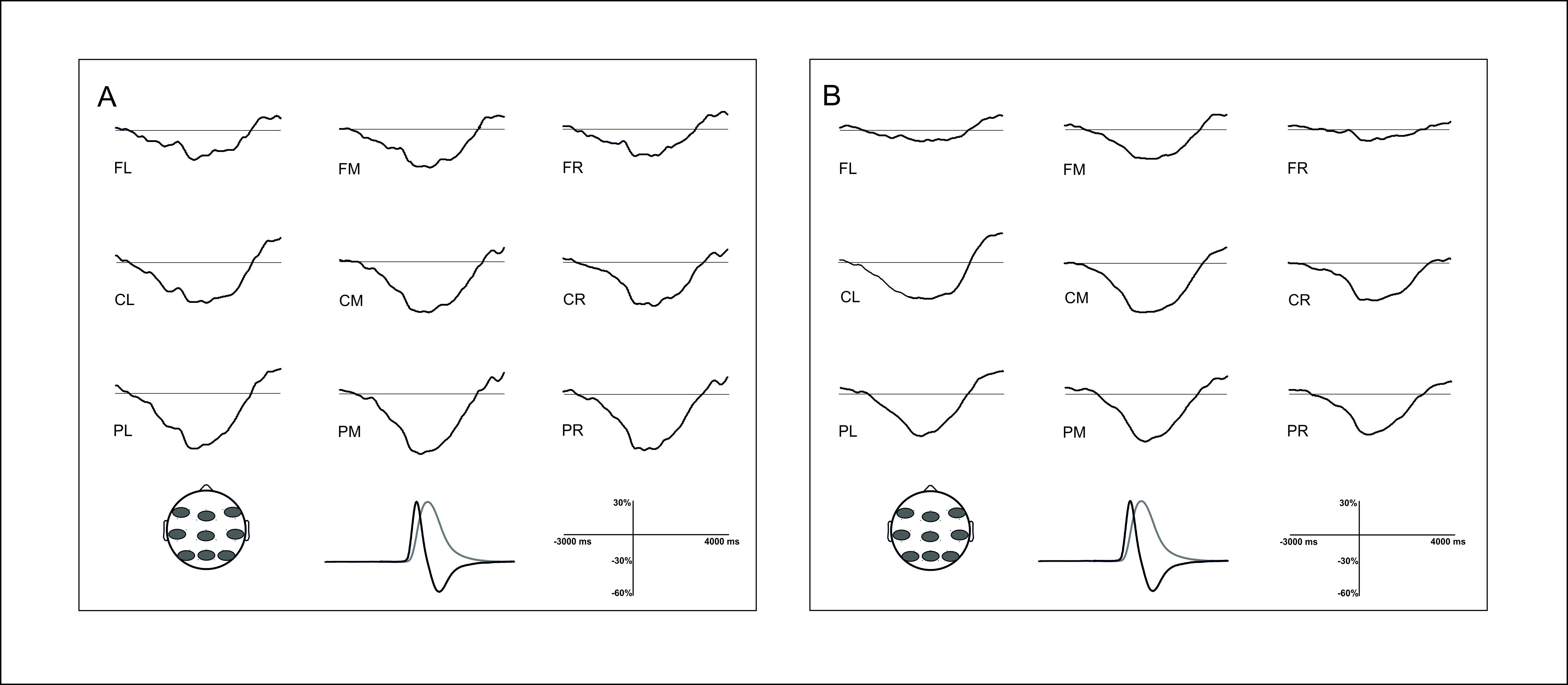


**Figure S2**


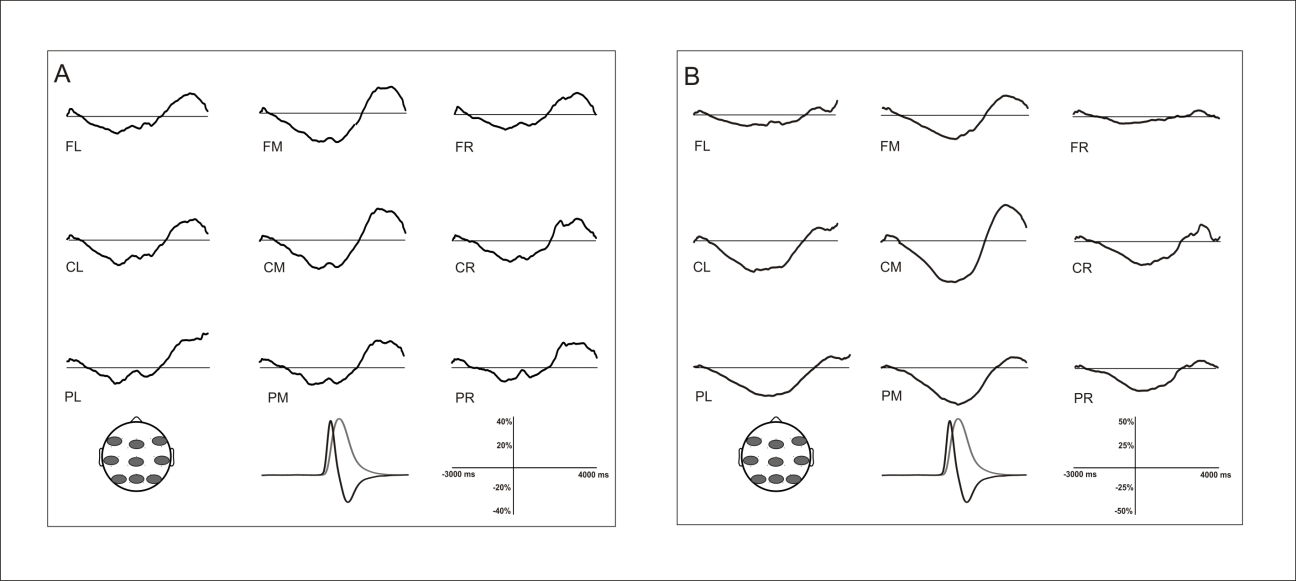


**Figure S3**


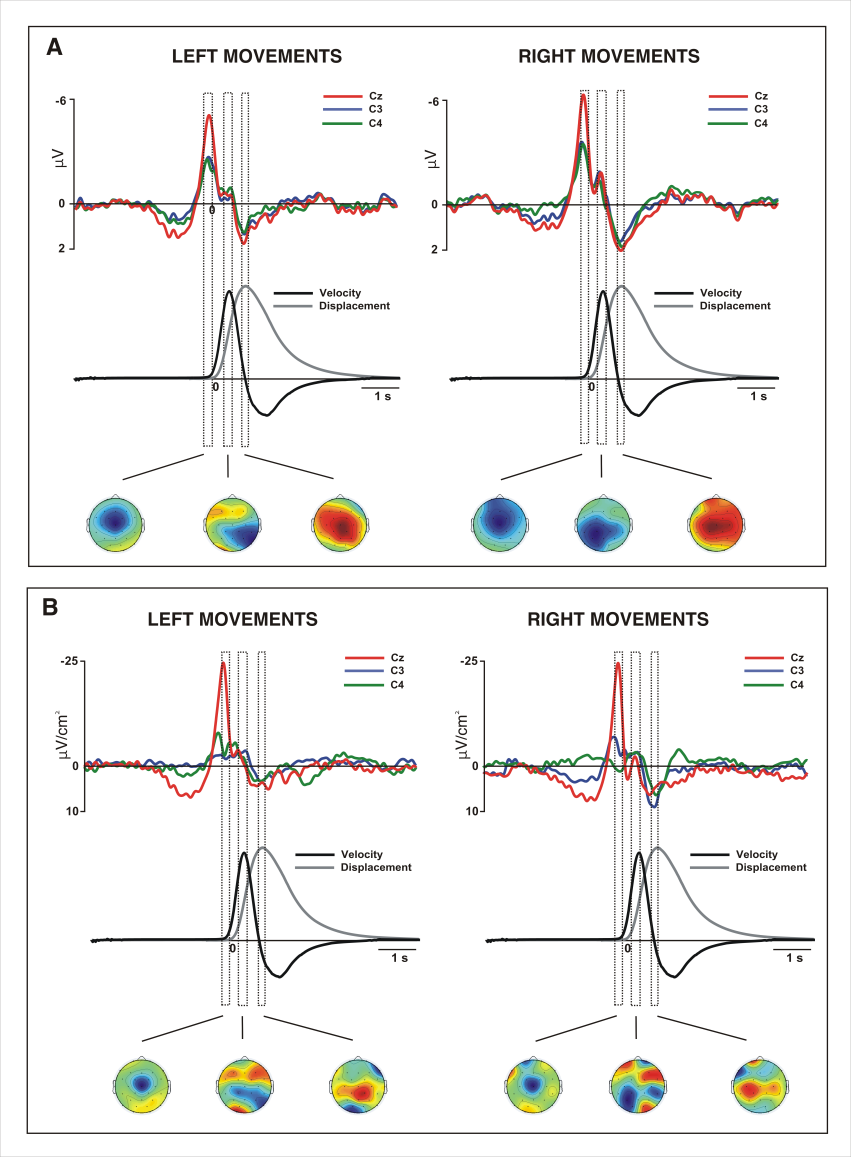


**Figure S4**


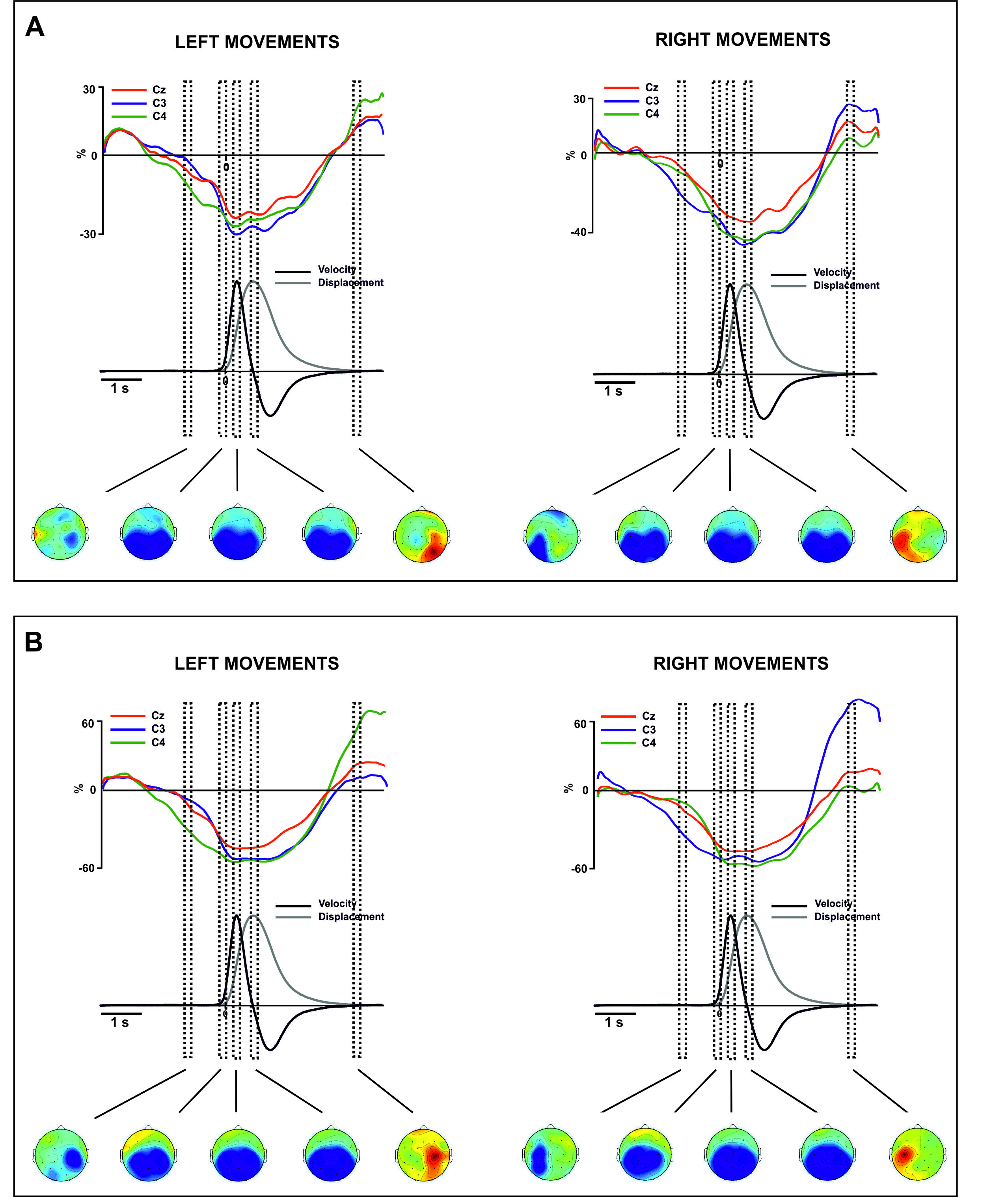


**Figure S5**

**
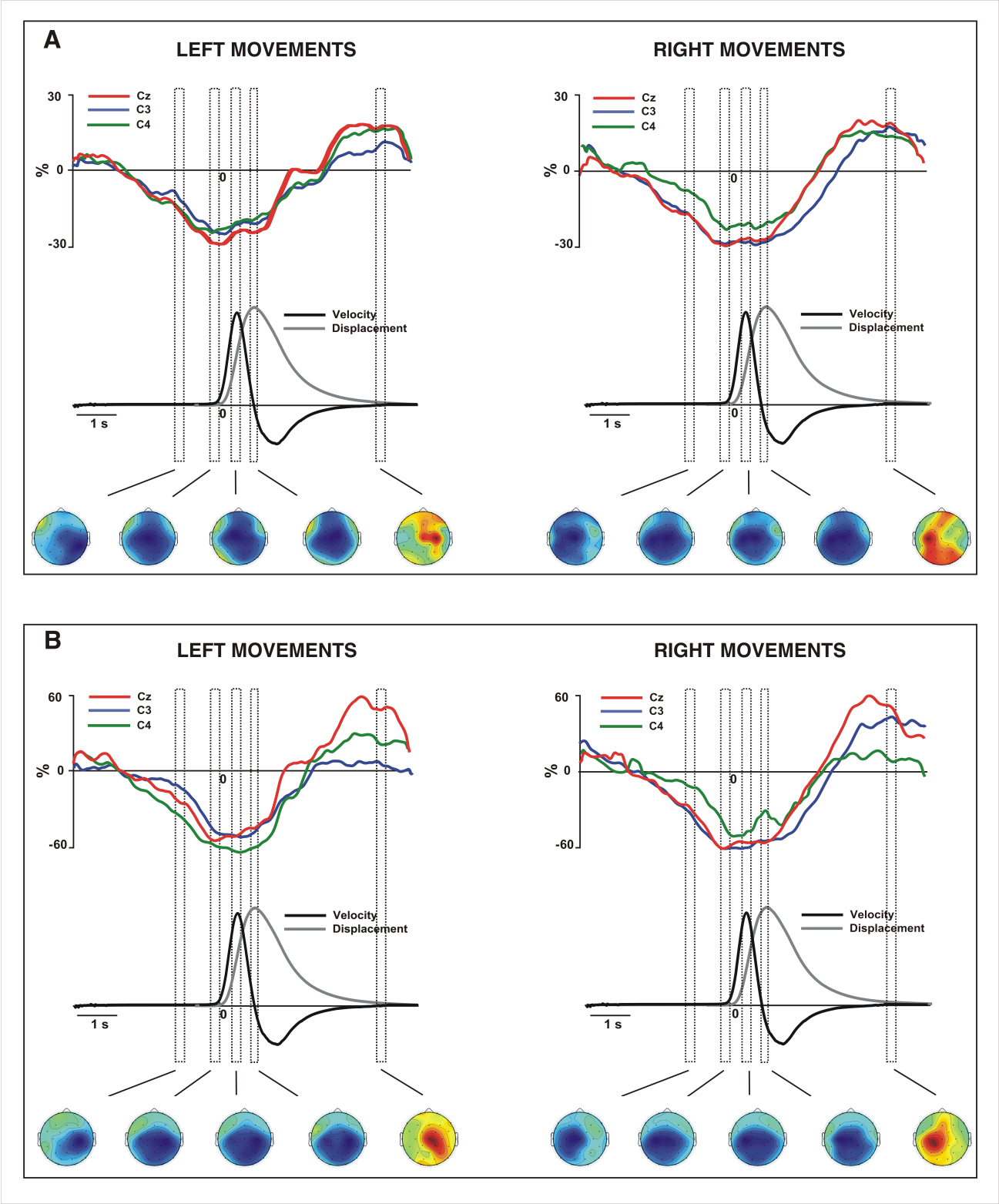
**

**Figure S6**

| **Table 1** |  |  |  |
| --- | --- | --- | --- |
| **Parameters (units)** |  |  |  |
|  | ***Left*** | ***Right*** | ***Merged*** |
| **MT (ms)** | 784 (206) | 777 (151) | 772 (131) |
| **Vmax (mm/s)** | 571 (247) | 510 (162) | 550 (180) |
| **T_Vmax (ms)** | 319 (40) | 310 (31) | 308 (22) |
| **T_Vmax/MT (%)** | 42.6 (6.1) | 41.3 (6.5) | 42.1 (5.9) |
| **Hmax (mm)** | 580 (380) | 522 (362) | 570 (370) |
| **T_Hmax (ms)** | 463 (286) | 434 (249) | 455 (203) |
| **T_Hmax/MT (%)** | 57.7 (8.1) | 54.3 (7.3) | 55.6 (6.5) |

Mean and S.E.M. of the behavioral parameters for left and right arm movements, and after merging data from both arms. Data did not reveal differences as a function of the acting arm. (MT: movement time; Vmax: maximal velocity; T_Vmax: Time when the maximal velocity is achieved; T_Vmax/MT: Percentage of the acceleration time; Hmax: Maximal altitude; T_Hmax: Time when the maximal altitude is achieved; T_Hmax/MT: Percentage of acceleration time for the altitude.
